# The potential role of gut microbiota outer membrane vesicles in colorectal cancer

**DOI:** 10.3389/fmicb.2023.1270158

**Published:** 2023-11-07

**Authors:** Ran Meng, Minmin Zeng, Ying Ji, Xinxiang Huang, Min Xu

**Affiliations:** ^1^Department of Gastroenterology, Affiliated Hospital of Jiangsu University, Zhenjiang, Jiangsu, China; ^2^Department of Biochemistry and Molecular Biology, School of Medicine, Jiangsu University, Zhenjiang, Jiangsu, China; ^3^Institute of Digestive Diseases, Jiangsu University, Zhenjiang, Jiangsu, China

**Keywords:** colorectal cancer, gut microbiota, outer membrane vesicles, gut homeostasis, immune regulation

## Abstract

Colorectal cancer (CRC) is a common malignant digestive tract tumor in colorectal regions. Considerable evidence now shows that the gut microbiota have essential roles in CRC occurrence and development. Most Gram-negative bacteria release outer membrane vesicles (OMVs) *via* outer membrane blistering, which contain specific cargoes which interact with host cells *via* intercellular communications, host immune regulation, and gut microbiota homeostasis. Studies have also shown that OMVs selectively cluster near tumor cells, thus cancer treatment strategies based on OMVs have attracted considerable research attention. However, little is known about the possible impact of gut microbiota OMVs in CRC pathophysiology. Therefore, in this review, we summarize the research progress on molecular composition and function of OMV, and review the microbial dysbiosis in CRC. We then focus on the potential role of gut microbiota OMVs in CRC. Finally, we examine the clinical potential of OMVs in CRC treatment, and their main advantages and challenges in tumor therapy.

## 1. Introduction

Colorectal cancer (CRC) is a common malignant digestive tract tumor. Currently, CRC has the world’s third highest new cancer incidence rates, and is one of the major life-threatening cancers (Siegel et al., 2020; Miller et al., 2022). In recent years, overall CRC incidence rates have increased in individuals under 50, which is a worrying trend (Spaander et al., 2023). At present, surgery, radiotherapy, and chemotherapy are the main CRC treatment methods. Additionally, targeted therapies and immunotherapies have generated some therapeutic effects in metastatic CRC (Kuipers et al., 2015). However, these new treatment options have had limited impact on CRC cure rates and long-term survival.

The human gut is a complex ecosystem where trillions of microorganisms (bacteria, fungi, and viruses) live. These microflora exert a significant impact on human health and disease (Fan and Pedersen, 2021). Considerable evidence now suggests that the gut microbiota is strongly associated with CRC development and prognosis outcomes (Meng et al., 2018; Wong and Yu, 2019; Song et al., 2020; Liu et al., 2022c). Microbiota dysbiosis modulates host metabolic and immune pathways, disrupting intestinal homeostasis, and promoting cancer development and progression (Weiss and Hennet, 2017; Gopalakrishnan et al., 2018). Also, modulating the gut microbiota and reversing established microbial dysbiosis may help prevent and treat CRC (Fong et al., 2020).

In the last decade, research interest in bacterial extracellular vesicles (BEVs) has increased exponentially, with BEV-based cancer therapies attracting major attention in the biomedical field (Chronopoulos and Kalluri, 2020; Sartorio et al., 2021). Of these, outer membrane vesicles (OMVs) are spherical, bilayered nanostructures released by Gram-negative bacteria, and are natural bacterial component carriers. These vesicles regulate intercellular communications by transferring lipopolysaccharide (LPS), lipids, proteins, nucleic acids, and toxins (Nagakubo et al., 2019; Toyofuku et al., 2019, 2023). Gut microbiota-derived OMVs, as key mediators of bacteria-host interactions, have reportedly multiple functions, including but not limited to, horizontal gene transfer (Domingues and Nielsen, 2017), immune response modulation (Kaparakis-Liaskos and Ferrero, 2015), regulating intestinal epithelial barrier integrity, and gut homeostasis (Díaz-Garrido et al., 2021). These properties provide specific and unique advantage in cancer diagnostics, vaccine development, targeted drug delivery, and other clinical tumor treatments (Chronopoulos and Kalluri, 2020; Sartorio et al., 2021).

In recent years, the pathological impact of BEVs on gastrointestinal cancers has been partially revealed. BEVs and contents have been promoted as biomarkers for early gastrointestinal cancer diagnosis (Barteneva et al., 2017). However, specific mechanisms and potential effects underlying interactions between gut microbiota OMVs and CRC remain unclear. Therefore, we reviewed research progress in OMV composition and function, and summarize the gut dysbiosis in CRC. Then, from a microbiota-host communications perspective, we focused on potential roles and related mechanisms of gut microbiota OMVs in regulating CRC progression. Finally, OMV prospects and limitations for CRC diagnosis and treatment were discussed.

## 2. Gut microbiota associated with CRC

Gut microbiota refers to about 40 trillion microbes living in the human intestinal tract. Normal gut microbiota have vital roles in gut homeostasis, host nutrient metabolism, immune regulation, and pathogen defenses. In contrast, gut microbiota dysbiosis exerts adverse effects on human health, resulting in many chronic diseases (Jandhyala et al., 2015; Chen et al., 2021; Liu et al., 2022c).

A growing body of evidence suggests a link between dysbiosis of the human gut microbiota and colorectal cancer. The gut microbiota of CRC patients showed Intestinal dysbiosis with lower abundance of potentially protective taxa and increased abundance of procarcinogenic taxa compared to healthy individuals (Gagnière et al., 2016; Yu et al., 2017; Wong and Yu, 2019). High-throughput microbiome sequencing has indicated consistent correlations between CRC and several specific bacteria, such as *Fusobacterium nucleatum*, *Escherichia coli*, *Bacteroides fragilis*, and *Enterococcus faecalis* were increased in CRC patients, whereas *Bifidobacterium*, *Lactobacillus*, and *Faecalibacterium prausnitzii* were absent (Zou et al., 2018; Table 1). For example, Liu et al. used 16S ribosomal RNA (rRNA) gene amplicon sequencing to analyze the composition of the gut microbiota in healthy controls, polyp patients, and colorectal cancer patients. They found that *Bacteroidetes*, *Firmicutes*, and *Proteobacteria* were enriched in CRC patients compared to healthy individuals, and many other researchers have come to the same conclusion (Liu et al., 2020). This study not only confirmed the significant differences in diversity and composition between the microbiota of CRC patients and healthy controls, but also found that the proportion of microorganism composition varies at different stages of CRC development. In addition, metagenomic studies in humans have identified novel associations between CRC and other bacteria. By performing metagenome-wide association studies on fecal samples, Yu et al. not only confirmed the known association of *Fusobacterium nucleatum* and *Peptostreptococcus anaerobius* with CRC, but also identified other members of specific microorganisms (such as *Parvimonas micra* and *Solobacterium moorei*) that are significantly associated with CRC (Yu et al., 2017).

**Table 1:** Gut microbiota dysbiosis in CRC.

**Microbe**	**Mechanism**	**References**
**Enriched in CRC**		
Enterotoxigenic *Bacteroides fragilis* (ETBF)	*B. fragilis* toxin production; promotion of inflammation	Wu et al. (2009), Chung et al. (2018), and Zamani et al. (2019)
*Fusobacterium nucleatum*	Promotion of inflammation; impairment of antitumor immunity, activation of β-catenin pathway	Castellarin et al. (2012) and Kostic et al. (2013)
*Escherichia coli*	Induction of a pro-inflammatory environment; genotoxin production(colibactin)	Arthur et al. (2012) and Iyadorai et al. (2020)
*Porphyromonas gingivalis*	Activation of NLRP3 inflammasome; promotion of CRC cells proliferation	Mu et al. (2020) and Wang et al. (2021a)
*Peptostreptococcus anaerobius*	Oxidative stress	Tsoi et al. (2017)
*Prevotella intermedia*	Promotion of inflammation	Dai et al. (2018)
*Parvimonas micra*	Promotion of inflammation	Yu et al. (2017) and Dai et al. (2018)
*Helicobacter pylori*	Promotion of inflammation	Teimoorian et al. (2018)
*Streptococcus gallolyticus*	Promotion of cell proliferation and angiogenesis	Abdulamir et al. (2011) and Aymeric et al. (2018)
*Enterococcus faecalis*	Activation of Wnt/β-catenin pathway; induction of transcription factors	Wang et al. (2017) and de Almeida et al. (2018)
**Depleted in CRC**		
*Bifidobacterium*	Butyrate production; reduction of pro-inflammatory cytokines	Kim et al. (2008) and Villéger et al. (2018)
*Lactobacillus*	T-cells activation; enhancement of antitumor immunity; mucus barrier maintenance	Chen et al. (2012)
*Faecalibacterium prausnitzii*	Butyrate producer; anti-inflammatory	Chen et al. (2012) and Shah et al. (2018)
*Roseburia*	Butyrate producer; anti-inflammatory	Wang et al. (2012)
*Eubacterium rectale*	Butyrate producer; inflammatory	Yu et al. (2017) and Zou et al. (2018)
*Clostridium butyricum*	Apoptosis of CRC cells; Immune homeostasis	Chen et al. (2015) and Dai et al. (2018)

In the imbalance of intestinal flora, pathogenic bacteria replace harmless commensal bacteria, causing inflammation in the host, promoting cell proliferation, and inducing carcinogenic signals and metabolites, thus contributing to the onset and progression of CRC. This process may occur through different mechanisms, including bacterial-derived genotoxins, microbial-derived metabolism, regulation of host defenses and inflammatory pathways, oxidative stress induction, and antioxidant defense regulation (Gagnière et al., 2016; Wang and Li, 2022). For example, *Fusobacterium* is one of the most common bacterial species in CRC tissues and metastases with CRC cells. *Fusobacterium nucleatum* has been shown to modulate several immune responses against colorectal cancer progression, induce the production of inflammatory mediators, and affect the tumor microenvironment (Castellarin et al., 2012). By using the Apc (Min/+) mouse model treated with human isolates of *F. nucleatum*, Kostic et al. (2013) found that *F. nucleatum* can generate a pro-inflammatory microenvironment by recruiting tumor-infiltrating myeloid cells, which is conducive for carcinogenesis. They also found that *F. nucleatum* can facilitate CRC progression by down-regulating anti-tumor T cell-mediated adaptive immunity (Kostic et al., 2013). In addition, related studies have found that colorectal carcinogenesis caused by *F. nucleatum* involves a variety of other mechanisms, including virulence factors, metabolism products, and miRNAs, and so on (Castellarin et al., 2012; Zhang et al., 2018).

Table 1 summarizes the changes in gut bacterial abundance and possible carcinogenic mechanisms in CRC. These studies have broadened our understanding of the role of gut microbiota in CRC pathogenesis.

## 3. Overview of bacterial OMVs

Under the background that the role of gut microbial communities in CRC occurrence and development has been widely studied, microbe-derived OMVs have emerged as novel players in the biomedical field. They were first isolated from Gram-negative bacteria (*Escherichia coli*) in the 1960s (Knox et al., 1967). Subsequently, they were observed in different Gram-negative bacteria, including *Vibrio cholerae*, *Salmonella typhimurium*, and *Pseudomonas aeruginosa* (Chatterjee and Das, 1967; Rothfield and Pearlman-Kothencz, 1969; Kadurugamuwa and Beveridge, 1995). Previous studies have reported that two main vesicle formation routes exist in Gram-negative bacteria: (1) OMV generation *via* outer membrane blebbing and (2) explosive cell lysis, where outer-inner membrane vesicles (OIMVs) and explosive outer-membrane vesicles (EOMVs) are formed (Toyofuku et al., 2019; Tulkens et al., 2020a).

**Figure 1:**
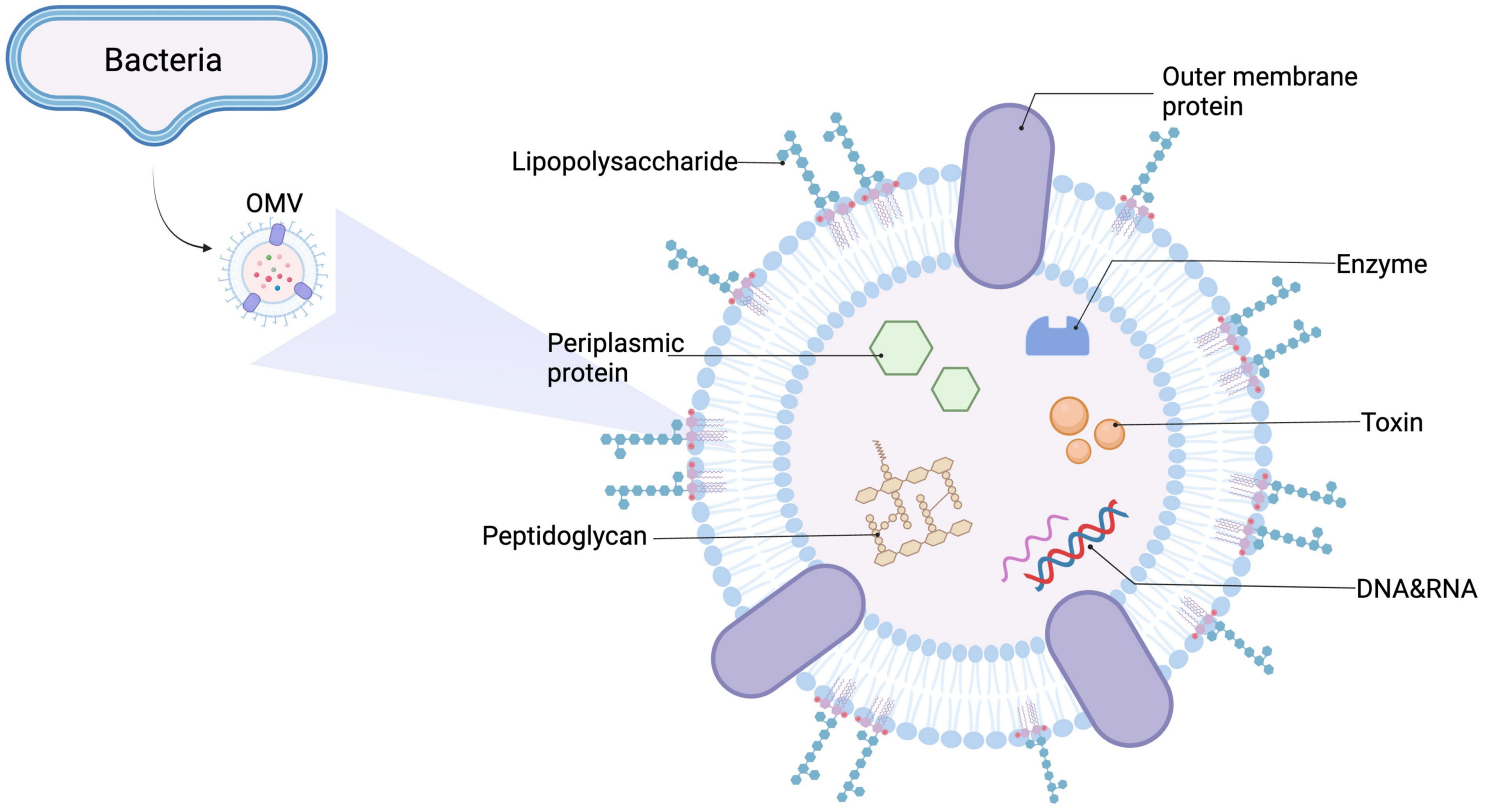
General outer membrane vesicles (OMVs) structure and content. OMVs are spherical, bilayered membrane nanostructures released by Gram-negative bacteria. They contain many biological elements from the parent bacterium, including lipopolysaccharide (LPS), periplasmic and membrane-bound proteins, enzymes, toxins, DNA, RNA, and other molecules.

In this review, we mainly focus on OMVs, which are spherical membrane particles measuring 20–300 nm in diameter. They are produced *via* outer membrane blebbing of gram-negative bacteria and are rich in outer membrane proteins and lipids, which act as multifunctional secretion and transport mechanisms for bacteria (Guerrero-Mandujano et al., 2017; Sartorio et al., 2021). Additionally, OMVs carry many cargo molecules, including membrane-bound and periplasmic proteins, LPS, nucleic acids (DNA and RNA), and toxins (Toyofuku et al., 2019; Chronopoulos and Kalluri, 2020). Both cargo quantity and content are affected by environmental and genetic factors, and vary among strains (Orench-Rivera and Kuehn, 2016; Volgers et al., 2018; Godlewska et al., 2019). The general OMV structure and composition is shown (Figure 1).

Nowadays, several mechanisms of OMV biogenesis have been proposed, including a loss of cross-linking between outer membrane (OM) and peptidoglycan (PG) layers, accumulated PG fragments or misfolded proteins in the periplasmic space, LPS remodeling, and bilayer couple modeling (Sartorio et al., 2021).

## 4. OMVs function in gut microbiota physiology

**Figure 2:**
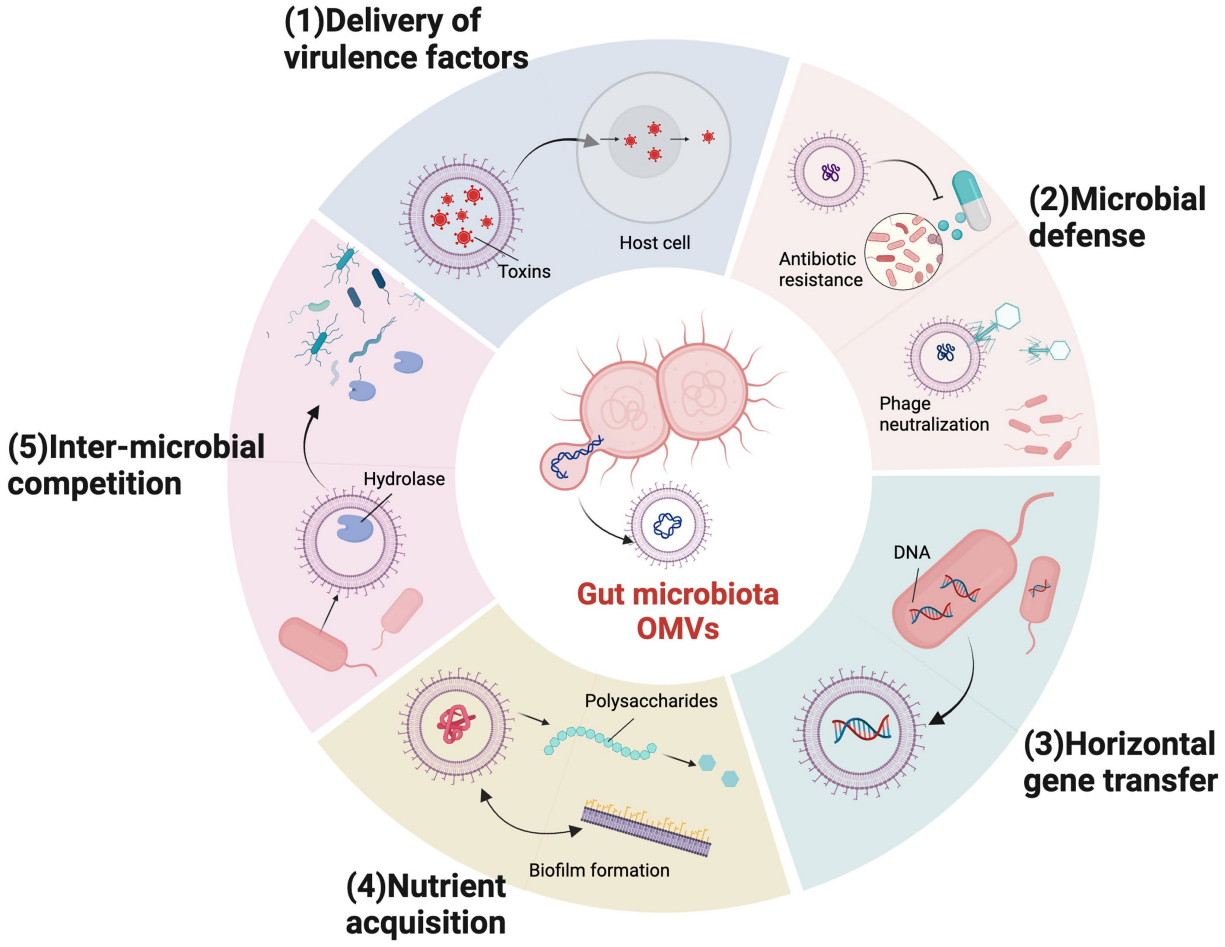
Outer membrane vesicles (OMVs) biological functions in gut microbiota. Bacterial OMVs have multiple biological functions required for survival and pathogenic mechanisms in gut microbial communities in the intestinal tract. (1) Virulence factor delivery. (2) Microbial defense mechanisms, including antibiotic deactivation and phage neutralization. (3) Horizontal gene transfer. (4) Nutrient acquisition *via* biofilm formation, polysaccharide catabolism, and iron acquisition. (5) Inter-microbial competition.

Specific OMV cargo molecules and biogenic pathways of different bacterial OMVs generate different biological functions. OMVs participate in intracellular and intercellular communications and mediate many physiological processes, including quorum sensing, gene transfer, toxin delivery, stress responses, microbiota homeostasis, pathogenesis, and immune regulation (Díaz-Garrido et al., 2021; Sartorio et al., 2021; Toyofuku et al., 2023; Figure 2).

OMVs are closely related to pathogenesis because they carry adhesins, toxins, and immunomodulatory compounds (Koeppen et al., 2016; Tian et al., 2023). OMVs directly deliver multiple virulence factors to the host cytoplasm and protect their contents from host lyase and antibodies, thus ensuring toxin activity (Macion et al., 2021). For example, the enterotoxigenic *Escherichia coli* (ETEC) secretes heat-labile enterotoxin *via* OMVs, resulting in strain pathogenicity (Horstman and Kuehn, 2000; Kesty et al., 2004). Similarly, uropathogenic *Escherichia coli* (UPEC) OMVs deliver cytotoxic necrotizing factor type 1 (CNF1), thereby attenuating neutrophil antimicrobial activity and chemotaxis (Davis et al., 2006). *S. typhimurium* also uses OMVs to transport virulence factors. Unlike other bacteria, they produce OMVs during intracellular life and escape the infected host cell. Then, bystander cells internalize OMVs containing cytolethal expansion toxin *via* dynamin-dependent endocytosis (Guidi et al., 2013).

In addition to toxin transfer, OMVs serve as microbial defense mechanisms contributing to immune evasion by bacteria. OMVs provide protection for parent bacteria against harmful substances, including antibiotics, phages, and reactive oxygen species (Díaz-Garrido et al., 2021). Horizontal gene transfer through OMVs can facilitate antimicrobial resistance in bacteria (Domingues and Nielsen, 2017). In 2011, OMVs were first identified as antimicrobial resistance gene carriers, and by extension resistance mechanisms, by Rumbo et al. The authors demonstrated a horizontal carbapenem resistance gene transfer mechanism *via* OMV release in *Acinetobacter baumannii* (Rumbo et al., 2011). Also, as OMVs have similar surface structures to the bacteria they originate from, they can neutralize phage and prevent direct interactions with bacterial cells (Azam and Tanji, 2019). For example, *V. cholerae* OMVs acted as natural decoys against three unique virulent phages (ICP1, ICP2, and ICP3) and formed defenses protecting bacteria from phage predation (Reyes-Robles et al., 2018).

OMVs also facilitate bacterial nutrient acquisition which may serve as a mechanism for competition with other bacteria. Biofilm formation protects diverse bacterial communities and facilitates nutrient acquisition and survival (Flemming et al., 2016), with evidence now suggesting that OMVs are essential in biofilm formation and stabilization (Wang et al., 2015; Brameyer et al., 2018; Begić and Josić, 2020). For example, Seike et al. (2020) observed that OMVs released by *Aeromonas* strains dose-dependently promoted biofilm formation. Some OMVs are rich in hydrolytic enzymes, such as *Bacteroidetes* OMVs, which break down complex polysaccharides in the intestinal lumen into simple carbohydrates, thus facilitating nutrient acquisition for microbial communities (Elhenawy et al., 2014; Liang et al., 2022). Other studies have shown that OMVs from different species contain iron acquisition proteins and bacterial cell surface receptors that recognize heme groups, suggesting pivotal roles for OMVs in iron acquisition in different bacteria (Schwechheimer and Kuehn, 2015). In a recent study, Dhurve et al. (2022) found the presence of TonB-dependent transporters (TonRs) capable of transporting various nutrients in the outer membrane vesicles (OMVs) of *Acinetobacter baumannii* DS002. They capture and transport ferric iron complexed with enterobactin into *A. baumannii* DS002 cells. In addition to the TonRs, *A. baumannii* OMVs also carry proteins with pathogenesis, immune evasion, and biofilm formation (Dhurve et al., 2022).

In terms of microbial competition, some OMVs carry degradative enzymes (lipases and endopeptidases) which kill other bacteria. Evidence exists that some OMVs in the *Myxococcus xanthus* contain antimicrobial factors and hydrolases, which lyse target bacterial cells and enhance predation activity (Marshall and Whitworth, 2019).

## 5. Gut microbiota OMVs interaction with host cells

Since microbial access to the intestinal epithelium is limited by the inner mucin layer, host communications mainly depend on microbiota-secreted factors (metabolites, proteins, and vesicles) which cross mucin layers and reach intestinal mucosa surfaces (Díaz-Garrido et al., 2021). Currently, growing evidence suggests that OMVs from gut microbiota origins are effective communication vehicles between the gut microbiota and hosts. OMVs produced by gut bacteria are detected in human body fluids and fecal samples and may critically influence host pathophysiology by regulating the gut microenvironment (Villard et al., 2021).

The main mechanisms whereby gut bacterial OMVs interact with human host cells include, OMV internalization by host cells, host immune response modulation, and the maintenance of gut environmental homeostasis (Kaparakis-Liaskos and Ferrero, 2015; Chronopoulos and Kalluri, 2020; Díaz-Garrido et al., 2021; Sartorio et al., 2021).

In addition, many studies support the beneficial effects of probiotics in CRC prevention and treatment (Ryu et al., 2022; Saeed et al., 2022; Huang et al., 2023). For CRC, the most common probiotics are *Lactobacillus* and *Bifidobacterium*, including other genera such as *Enterococcus*, *Streptococcus* and *Leuconostoc* (Zou et al., 2018; Wong and Yu, 2019). Like OMVs, probiotics can also regulate the gut microbiota through immunomodulation, inhibition of pathogenic bacteria colonization and enhancement of intestinal barrier function, thus improving host health (Liu et al., 2016). Importantly, some of these probiotics, such as *Escherichia coli* Nissle 1917 (ECN) and the intestinal commensal strain ECOR63, are able to protect the physical and biological barriers of the gut through the production of OMVs in addition to the secreted factors (the secreted protein TcpC) (Alvarez et al., 2016, 2019). Taking cues from these studies, probiotics may also be developed to modulate host intestinal function, providing new insights into the combined application of probiotics and OMVs for CRC management.

We examine the above perspectives and fouse on OMV interactions between the gut microbiota and the host.

### 5.1. OMVs internalization by host cells

As a broad secretory pathway in Gram-negative bacteria, OMVs enter host cells and release many cargoes. This process may occur through different pathways depending on OMV properties (Barteneva et al., 2017; Sartorio et al., 2021). Currently, three main OMV internalization pathways are proposed: (1) clathrin-mediated endocytosis, (2) clathrin-independent endocytosis, and (3) direct membrane fusion (Odonoghue and Krachler, 2016; Liu et al., 2022a).

Endocytosis is the primary OMV internalization pathway into host cells. Endocytic pathways are divided into clathrin-mediated and clathrin-independent endocytosis, including lipid raft-mediated processes. Specific pathways depend on OMV dimensions and vesicle components (proteins, toxins, or surface structures) which target specific receptors on host cell membranes (Sartorio et al., 2021). Clathrin-mediated endocytosis occurs *via* the formation of lattice-protein envelope depressions. In *Heliobacter pylori*, clathrin-mediated endocytosis is the primary host transport route for absorbing OMVs (Parker et al., 2010; Olofsson et al., 2014). In other gut microorganisms, OMVs derived from *E. coli* O157, non-O157 Enterohemorrhagic *E. coli* (EHEC), and nonpathogenic *E. coli* strains are also internalized by clathrin-mediated endocytosis (Bielaszewska et al., 2013; Kunsmann et al., 2015; Fábrega et al., 2016).

Other studies have suggested lipid raft roles in facilitating OMV entry. Lipid rafts are dynamic membrane microregions rich in selected lipids and proteins (Díaz-Garrido et al., 2021). Lipid raft-mediated endocytosis is primarily attributed to caveolin, e.g., ETEC endocytosis depends on cholesterol-rich lipid rafts. Entering vesicles are partially colocalized with caveolin, delivering active enterotoxins and other bacterial envelope components into host cells (Kesty et al., 2004). Alternatively, lipid rafts mediate endocytosis independent of caveolin and dynein; OMVs from *Campylobacter jejuni* are delivered to intestinal epithelial cells (IECs) *via* this caveolin-independent lipid raft-mediated endocytosis pathway, thereby inducing innate immune responses in IECs (Elmi et al., 2012).

Direct membrane fusion also allows OMV entry into cells (Odonoghue and Krachler, 2016). Several studies used dye labeling techniques to show that OMV fusion with eukaryotic membranes usually occurred in lipid raft domains. OMV fusion with host cell lipid rafts induced actin remodeling, allowing direct soluble cargo diffusion into the host cytoplasm (Rompikuntal et al., 2012; Jäger et al., 2015; Bielaszewska et al., 2017). However, this internalization mechanism is rarely found in gut microbiota OMVs (Barteneva et al., 2017).

### 5.2. Gut microbiota OMVs regulation of host immune responses

**Figure 3:**
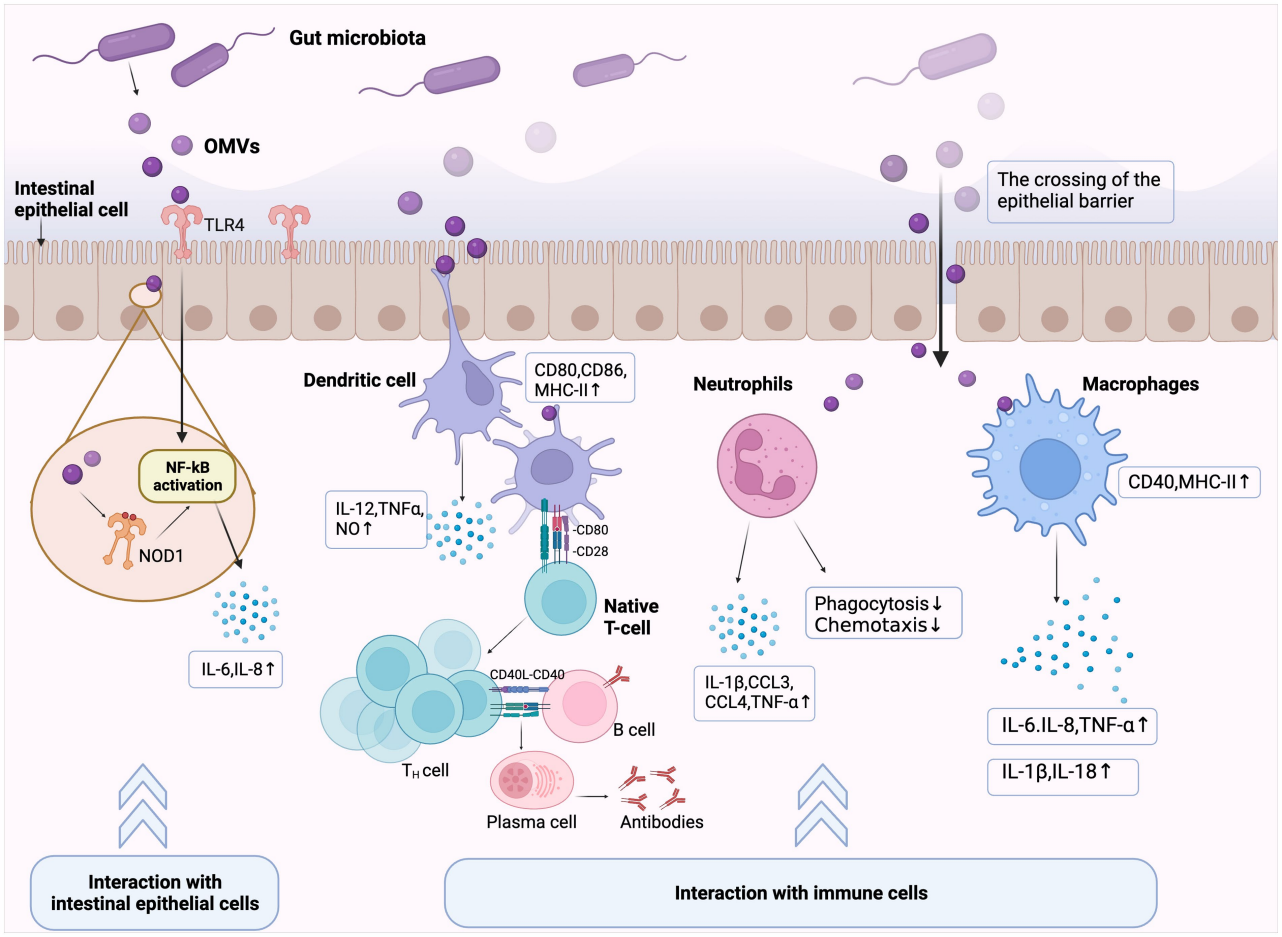
Immunomodulatory effects elicited by gut microbiota outer membrane vesicles (OMVs). Gut microbiota OMVs cross the mucosal barrier and exert immunomodulatory effects through two main pathways. (1) Interactions with intestinal epithelial cells (IECs): OMVs internalized by IECs activate extracellular Toll-like or cytoplasmic Nucleotide oligomerization domain (NOD)-like receptors, which induce or limit immune effector production by IECs, and then initiate innate immune responses. (2) Interactions with immune cells: OMVs cross the intestinal epithelial barrier and translocate to the submucosa, thereby activating intestinal immune cells, including lymphocytes, macrophages, and dendritic cells. OMVs promote antigen presentation and further initiate host adaptive immune responses by inducing or inhibiting chemokine production in cells and causing gut homeostatic/pathological states.

Gut bacteria OMVs carry multiple microbial associated molecular patterns, including LPS, lipoteichoic acid (LTA), PG, bacterial DNA, and RNA. These patterns activate pattern recognition receptors (PRRs) in IECs and immune cells, thereby initiating host pro-inflammatory signaling cascade responses or immune tolerance. PRR induction mechanisms and immunomodulatory effects from different OMVs also differ and depend on the uptake pathway and bacterial ligand (Kaparakis-Liaskos and Ferrero, 2015; Vanaja et al., 2016; Mancini et al., 2020). In this section, we discuss OMV function in regulating host immune responses from two perspectives: gut microbiota OMVs interaction with host IECs and immune cells (Figure 3).

#### 5.2.1. OMVs interaction with IECs

The intestinal epithelial barrier is the host’s first line of defense. OMVs interact directly with IECs and stimulate host PRRs [e.g., Toll-like receptor 4 (TLR4)] *via* several immune-related molecules, thereby inducing or limiting epithelial cell cytokine and chemokine production (Gu et al., 2019b; Díaz-Garrido et al., 2021).

TLR proteins on host cell surfaces recognize invading microbial components and activate immune response, which have key roles in the innate immune system (Zhai et al., 2018). TLR2 and TLR4 recognize OMV surface ligands, such as LPS, LTA, and PG. *E. coli* OMVs are enriched in LPS, which induce TLR4-dependent pro-inflammatory responses. Additionally, other *E. coli* OMVs membrane components, such as OmpW, elicited pro-inflammatory responses independent of TLR4 and Ca^2+^ signal transduction (Söderblom et al., 2005; Bielaszewska et al., 2018). Intracellularly, immune receptors, such as nucleotide-binding oligomerization domain-like receptor 1 (NOD1) and nucleotide-binding oligomerization domain-like receptor 2 (NOD2), bind bacterial PG fragments and are important in host defenses and inflammatory diseases (Kaparakis-Liaskos and Ferrero, 2015; Trindade and Chen, 2020). Internalized bacterial OMVs (containing PG fragments) by IECs also activate NOD receptors, which trigger immune effector secretion and initiate innate immune responses. This process is crucial for maintaining intestinal environmental stability and microbiota homeostasis (Irving et al., 2014; Chu et al., 2016). In *H. pylori*, OMVs induce pro-inflammatory signals in human cells and mouse embryonic fibroblasts through a NOD1-dependent but TLR-independent mechanism, thereby promoting host inflammation and pathology. This finding identified OMVs as a generalized mechanism whereby Gram-negative bacteria deliver peptidoglycan to cytosolic NOD1 in host cells (Kaparakis et al., 2010). Similarly, Cañas et al. (2018) reported that OMVs isolated from probiotic and commensal *E. coli* strains activated NOD1 signaling in IECs, thereby inducing IL-6 and IL-8 expression.

In addition to initiating the inflammatory response in IECs, OMVs can promote bacterial survival in the host by limiting inflammation (Kaparakis-Liaskos and Ferrero, 2015). For example, *H. pylori* OMVs induces anti-inflammatory factor IL-10 and pro-inflammatory factor IL-6 production in host cells, regardless of donor infection status (Winter et al., 2014; Chmiela et al., 2018). This suggested a role for *H. pylori* OMVs in stimulating innate pro-inflammatory and anti-inflammatory responses. Furthermore, human gastric epithelial cell stimulation with different *H. pylori* OMV doses elicited different host inflammatory responses (Ismail et al., 2003). Thus, OMV interactions with the intestinal epithelium may enhance the host immune response, or conversely, downregulate immune reactivity to support infection persistence.

#### 5.2.2. Direct OMVs interaction with immune cells

When OMVs cross the IEC barrier to the submucosa, immune cells in gut-associated lymphoid tissue, such as dendritic cells (DCs), macrophages, and neutrophils, can interact directly with these OMVs. Subsequently, OMVs initiate host adaptive immune responses by upregulating/inhibiting these immune cells activation and cytokine and/or chemokine production (Kaparakis-Liaskos and Ferrero, 2015; Díaz-Garrido et al., 2021).

DC maturation is required to initiate antigen-specific immune responses. Growing evidence now suggests that bacterial OMVs induce DC maturation and cytokine production. *S. typhimurium* OMVs reportedly induced CD86 and MHC-II expression on DC surfaces and enhanced the pro-inflammatory mediator expression of nitric oxide, tumor necrosis factor (TNF)-α, and IL-12. They also stimulated protective B and T cell responses *in vivo* (Alaniz et al., 2007). Similarly, OMVs from the probiotic *Escherichia coli* Nissle 1917 (EcN) and commensal *Escherichia coli* strains activated DCs in a strain-specific manner to promote T cell subset differentiation (Diaz-Garrido et al., 2019, 2022). Additionally, Shen et al. reported that *Bacteroides fragilis* released polysaccharide (PSA) in OMVs which induced immunomodulatory effects. DCs sensed these PSA-associated OMVs *via* TLR2, then enhanced regulatory T cells and produced the anti-inflammatory cytokine IL-10 (Shen et al., 2012). Thus, OMVs released by gut microbiota help induce DC maturation, facilitated antigen presentation, and help bridge innate and adaptive immune responses.

Macrophages are also important players in OMV-mediated inflammatory responses (Guangzhang et al., 2023; Imamiya et al., 2023). One study reported that OMVs from *Shigella flexneri* increased major histocompatibility complex MHCII and CD40 levels in macrophages when co-cultured with macrophages (Pastor et al., 2017). Additionally, OMVs modulated neutrophil function in different ways, e.g., UPEC OMVs contain CNF1 which attenuates neutrophil phagocytosis and chemotaxis (Davis et al., 2006). In contrast, a recent study showed that low doses of gut microbiota OMVs enhanced pro-inflammatory sensitivity in murine neutrophils and induced adaptive responses in neutrophils *in vitro* (Lajqi et al., 2022).

However, the regulatory mechanisms whereby OMVs initiate host adaptive immunity remain unclear. Firstly, innate immunity regulation by OMVs may be important influencing factors. OMV antigens are delivered by antigen-presenting cells to T cells and inducing antigen-specific B cell responses. Secondly, recent studies reported that differential immunomodulatory effects by OMVs in gut microbiota were partially mediated *via* miRNA (microRNA) regulation (Díaz-Garrido et al., 2020). miRNAs are post-transcriptional regulators of the immune system and help fine-tune several signaling pathways (Belcheva, 2017). Thus, differential miRNA expression mediated by OMVs could exert different effects on host immune responses.

### 5.3. The effects of gut microbiota OMVs on intestinal barrier integrity

Intestinal epithelial barrier integrity is essential for maintaining intestinal dynamic balance. It provides a selective permeable barrier that limits the penetration of harmful luminal molecules (e.g., pathogens, toxins, and antigens), without interfering with appropriate nutrient and water absorption activities. Disruption to this permeable barrier increases intestinal permeability and harmful molecule infiltration into the lumen, causing immune system disorder and disease in the gut (Luissint et al., 2016; Takiishi et al., 2017; Lin et al., 2022). Epithelial barrier integrity is facilitated by intercellular tight junction (TJ) structures. Different transmembrane proteins, such as occludin, claudins, cytosolic scaffold proteins, and zonula occludens (ZOs), are important proteins maintaining TJ function. Additionally, other structures help maintain connections between IECs, such as adherens junctions, gap junctions, and desmosomes (Suzuki, 2013; Sartorio et al., 2021). Studies have suggested that microbiota OMVs may significantly impact intestinal epithelial barrier function *via* TJ protein or host inflammatory response regulation. Moreover, their regulatory effects may vary depending on their origin; OMVs produced by most pathogens impair intestinal barrier function, a property closely related to parent bacteria. In contrast, OMVs secreted by intestinal commensal and probiotic bacteria mostly strength epithelial barrier integrity and suppress intestinal inflammation levels (Tulkens et al., 2020b; Ghosh et al., 2021).

#### 5.3.1. OMVs maintain intestinal epithelial barrier integrity

Studies have shown how OMVs enhance epithelial barrier integrity. OMVs from some intestinal commensal bacteria can exert barrier protection by increasing TJ-related protein expression (Shen et al., 2012; Chelakkot et al., 2018; Hiippala et al., 2018).

In the IEC lines T-84 and Caco-2, OMVs from the probiotic *Escherichia coli* Nissle 1917 (EcN) and the intestinal commensal strain ECOR63 enhanced the intestinal barrier and reduced intestinal permeability by upregulating ZO-1 and claudin-14 and downregulating claudin-2. Furthermore, they both activated compensatory regulatory mechanisms affecting TJ protein transcriptional and post-transcriptional levels, thereby protecting against enteropathogenic *E. coli*-induced intestinal epithelial barrier dysfunction (Alvarez et al., 2016, 2019). Similarly, OMVs produced by the intestinal commensal bacterium *Akkermansia muciniphila* improved intestinal permeability by enhancing TJ function. In a high-fat diet-induced diabetic mouse model, *A. muciniphila*-derived OMVs neutralized increased intestinal permeability in LPS-treated Caco-2 cells. Western blotting showed that vesicles upregulated TJ protein expression, including occludin, ZO-1, and claudin-5. Interestingly, *A. muciniphila* OMVs had more substantial regulatory effects when compared with live bacteria (Chelakkot et al., 2018). The authors suggested that these OMVs could be used to positively modulate intestinal barrier integrity, reduce inflammation, and provide therapeutic potential for inflammatory disease. Several studies reported other mechanisms whereby probiotic EcN OMVs protected intestinal barrier function, e.g., in human colonic explants, EcN OMVs activated IL-22 expression, which helped maintain an intact epithelial barrier by inducing mucin production in goblet cells (Fábrega et al., 2017).

#### 5.3.2. OMVs disrupt the intestinal epithelial barrier

Conversely, OMVs may destroy epithelial barrier integrity and allow pathogen invasion into the intestinal epithelium. Firstly, OMVs can directly induce apoptosis in IECs. In macrophage/Caco-2 co-cultures, *Fusobacterium nucleatum* OMVs promoted epithelial barrier loss and oxidative stress damage, which were associated with epithelial necroptosis caused by receptor-interacting protein kinase 1 (RIPK1) and receptor-interacting protein kinase 3 (RIPK3) activation. This phenomenon was also verified in mouse colitis models (Newton, 2015; Liu et al., 2021).

Additionally, some OMV cargoes from intestinal pathogens may destroy connections and adhesion between IECs. In a recent study, OMVs from *E. coli* BL21 were incubated with Caco-2, HT29, and NCM460 cells, after which OMVs were internalized and released LPS into the host cytosol. Intracellular LPS activated caspase-5, which in turn, downregulated E-cadherin expression and caused intestinal barrier dysfunction (Wang et al., 2021b).

## 6. Potential role of gut microbiota OMVs on CRC

Although gut microbiota roles in CRC occurrence and progression have been widely studied, few studies have focused on microbial-derived OMV effects on CRC. For example, it has been shown that *Bacteroides fragilis* and its secreted toxin are associated with CRC carcinogenesis (Chung et al., 2018; Zamani et al., 2019), however, for *B. fragilis* OMVs, it is unclear if they affect CRC occurrence and progression. Thus, researchers are expected to pay more attention to the relationship between OMVs of intestinal bacterial origin and gastrointestinal cancer. Recently, Park et al. (2021) performed a metagenomic OMV analysis in stool samples from CRC patients and controls using 16S ribosomal DNA gene amplicon sequencing. The authors identified significant differences in microbial composition, homogeneity, and diversity in gut microbiota-derived OMVs from CRC patients when compared with controls. Additionally, OMV composition and diversity in stool from late-diagnosed CRC subjects were significantly changed when compared with early-diagnosed CRC subjects (Park et al., 2021). Therefore, gut bacterial origin OMVs may have essential regulatory roles in CRC development and progression, similar to gut bacteria. Although there are no comprehensive studies demonstrating that OMVs directly affect CRC development and progression, some studies on the interaction of OMVs with CRC cells or TME could provide hints for their putative role in CRC (Table 2).

**Table 2:** Possible gut microbiota outer membrane vesicles (OMVs) effects on colorectal cancer (CRC).

**OMVs source**	**Effect of OMVs**	**Mechanisms**	**Cell lines**	***In vivo***	**References**
**CRC promotion**					
*Vibrio cholerae*	Induce the expression of genes related to cell differentiation	Regulate gene transcription, induce epigenetic changes	HCT8	–	Vdovikova et al. (2018)
*Fusobacterium nucleatum*	Promote intestinal inflammation	Activate TLR4 and NF-κB	HT-29	–	Engevik et al. (2021)
	Generation of a pro-inflammatory environment	siglec-7 recognizes and combines LPS^2^ of OMVs	Human Primary Immune Cells	–	Lamprinaki et al. (2021)
**CRC inhibition**					
Attenuated *Escherichia coli* W3110△*msbB*	Suppress established tumors, prevent tumor metastasis	Mediate anti-tumor immune response by IFN-γ	B16-BL6, CT26, 4 T1, MC38	CT26 tumor-bearing mice	Kim et al. (2017)
	Reverse the immunosuppressive tumor microenvironment	Immune activation and checkpoint inhibition	B16-F10, CT26	CT26 tumor-bearing mice	Li et al. (2020b)
*Escherichia coli* Nissle 1917 (EcN)	Inhibit cell proliferation	Induce S/G2 cell cycle arrest and DNA damage	HT-29	–	Alvarez et al. (2019)
	Enhance intestinal epithelial barrier function	Up-regulate the expression of tight junction protein	T-84, Caco-2	–	Alvarez et al. (2016)
*Escherichia coli* BL21	Significantly inhibit tumor growth	Acts as immunostimulants for TME reprogramming	CT-26	–	Qing et al. (2020)
*Salmonella typhimurium*	Promote cells apoptosis, Inhibit tumor development	Up-regulate the expression of caspase-3, Beclin-1, and CD49b; down-regulate the expression of Ki-67 and VEGF^6^ gene	HTC116, MCF-7, HepG2	Ehrlich solid carcinoma-bearing mice	Aly et al. (2021)
	Decrease cancer cells viability	Exhibite cytotoxic effect on cancer cell	MCF-7, Caco-2	–	Aly et al. (2019)

### 6.1. CRC promotion by gut microbiota OMVs

Gut microbial OMVs may indirectly promote CRC progression by inducing inflammation and invoking tolerogenic immune reprogramming of the tumor microenvironment (TME) (Barteneva et al., 2017). OMVs interact with different cells in the TME. They induce vascular endothelial growth factor (VEGF) expression and the autocrine activation of its receptor, thus favoring cell proliferation and angiogenesis. Further, the distal action of systemic gut microbiota OMVs might also be related to the metastatic dissemination of tumors. With the continuous dysbiosis in gut microbiota, OMVs could interact with host cells in distant organs, initiate pro-inflammatory signaling, and trigger changes in the myeloid landscape, providing pre-metastatic niches for future colonization (Chronopoulos and Kalluri, 2020). Specific OMV components could been linked with regulatory functions affecting host epigenome and gene expression (Celluzzi and Masotti, 2016). Vdovikova et al. (2018) investigated the genomic and epigenetic consequences of *Vibrio cholerae* and nonpathogenic commensal *Escherichia coli* OMVs co-culture with CRC cells. The authors observed that OMVs increased gene expression associated with CRC cell differentiation, and further demonstrated a role for *V. cholerae*-derived membrane vesicles in promoting CRC differentiation through selective gene transcription (Vdovikova et al., 2018).

Gut microbiota OMVs may also promote CRC development by inducing host inflammatory responses. *F. nucleatum* in CRC patient guts was reportedly involved in CRC development by modulating innate immune cell. Engevik et al. (2021) reported that adding purified *F. nucleatum* OMVs to colonic epithelial cells activated TLR-4 and affected nuclear factor kappa B (NF-κB) signaling, thereby stimulating pro-inflammatory cytokine IL-8 and TNF production and promoting intestinal inflammation with respect to intestinal microbial depletion. Similarly, Lamprinaki et al. (2021) showed that *F. nucleatum* ssp. *Animalis* -derived OMVs or LPS induced pro-inflammatory profiles in human monocyte-derived DCs and tumor-associated profiles in human monocyte-derived macrophages by binding to Siglec-7, thereby generating a pro-inflammatory environment promoting CRC progression. Moreover, the authors discussed a new CRC therapy strategy targeting OMV-Siglec-7 interactions (Lamprinaki et al., 2021).

### 6.2. CRC inhibition by gut microbiota OMVs

In contrast, OMVs secreted by some gut microbiota have unique structures and immunostimulatory effects, which effectively induce anti-tumor immune responses in hosts, and have significant potential in CRC therapy. After *E. coli* W3110*msbB* mutant-derived OMVs injection into the tail vein of CRC CT26 mice, OMVs specifically targeted and accumulated in tumor tissue and subsequently induced substantial interferon gamma (IFN-γ) levels and promoted T cell-mediated immune responses, thereby suppressing established CRC and preventing tumor metastasis (Kim et al., 2017). Additionally, OMVs can improve antitumor efficacy by acting as powerful immune stimulators in TME reprogramming. Qing et al. used pH-sensitive calcium phosphate shells (CaP) on the surface of *E. coli* (BL21) OMVs, which enabled potent OMV-based TME reprogramming without side effects. Importantly, CaP shells facilitated acidic TME neutralization, leading to highly effective M2-to-M1 macrophage polarization for improved antitumor effects. After injecting OMVs into CRC mice, tumor growth was significantly inhibited (Qing et al., 2020).

In addition to acting as antitumor immune response stimulators, gut microbiota OMVs may exert antitumor effects by directly inducing CRC cell apoptosis. Aly et al. (2021) investigated *S. typhimurium* ATCC 14028 OMV (ST-OMV) effects on an *in vitro* human colorectal cancer cell line (HTC116) and a mouse model, and showed that ST-OMVs promoted apoptosis and reduced tumor invasion, with potential anti-CRC effects.

When combined, these studies suggest that gut microbiota OMVs, as bacterial-host communicators, have an important impact on CRC development and progression. Therefore, there is great potential for the development of innovative OMV-based CRC therapies.

## 7. Potential applications of gut microbiota OMVs for CRC treatment

**Figure 4:**
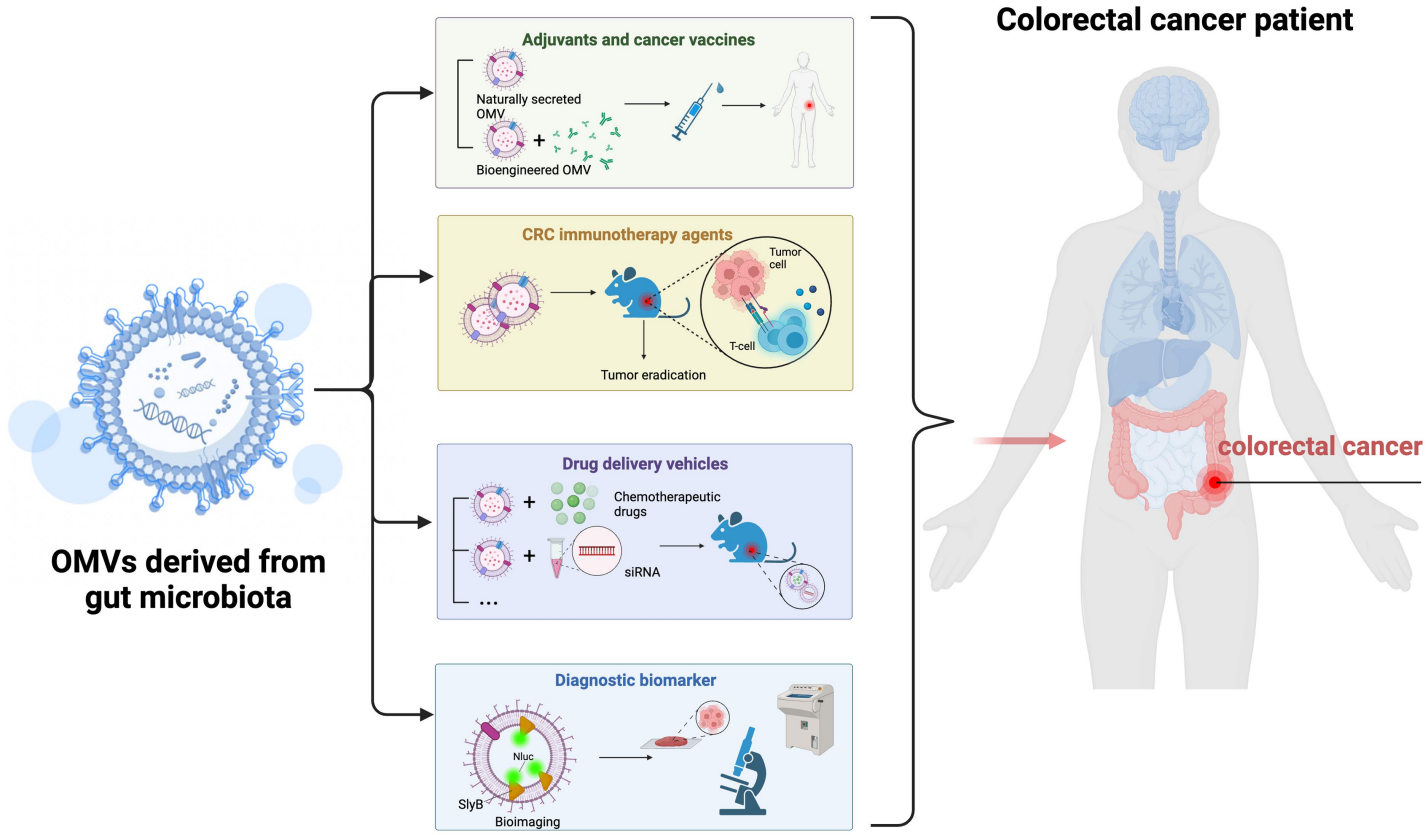
Possible gut microbiota outer membrane vesicles (OMVs) applications in colorectal cancer (CRC) treatment. (1) Gut microbiota-derived OMVs effectively activate host immune responses and may be ideal candidates for tumor vaccine and adjuvant development. (2) OMVs may be used as cancer immunotherapeutic agents to eradicate tumor tissue by inducing long-term antitumor immune responses. (3) OMVs may be used as drug delivery vehicles and undergo bio-engineering to enhance tumor-targeting. (4) Gut microbiota-derived OMVs may have essential roles as CRC diagnostic biomarkers.

In recent years, OMVs have received considerable attention as emerging cancer treatment strategies (Chronopoulos and Kalluri, 2020; Li et al., 2020a). Due to their many properties, in particular high levels of immunogenic components, they have tremendous advantages as antitumor immunotherapies and drug-targeted therapies (Xie et al., 2022; Won et al., 2023). Several studies have examined bacterial OMVs for CRC treatment. However, like many new therapeutic approaches, more prospective studies are required in this field. In this section, we discuss the possibility of gut microbiota OMVs in CRC treatments from several perspectives (Figure 4).

### 7.1. OMVs as adjuvant and tumor vaccines

Gut microbiota OMVs contain multiple antigens and pathogen-associated molecular patterns from bacterial sources, which effectively activate host immune responses. Therefore, OMVs can act as low-toxicity and efficient vaccines (Micoli and MacLennan, 2020; Balhuizen et al., 2021). Additionally, OMVs have other characteristics, including non-self-replication, <300 nm in size, and natural adjuvant activity (Gerritzen et al., 2017; Wang et al., 2022). Altogether, these characteristics make OMVs ideal candidates for tumor vaccine development.

It is accepted that DC-induced cytotoxic CD8+ T cells control tumor growth (Raskov et al., 2021). Therefore, using vaccines to stimulate antigen-specific CD8+ T cell responses in CRC could be a promising therapeutic strategy. Schetters et al. (2019) reported that *S. typhimurium* OMVs interacted with DCs to induce their maturation and thus activate CD8+ T host cell responses. The authors demonstrated that *S. typhimurium* OMVs could be developed as effective vaccines to inhibit tumor growth, with implications for CRC treatment. More importantly, genetic engineering techniques can be used to modify bacteria and purify recombinant OMVs as therapeutic CRC vaccines. Vesicle composition makes it easy to engineer modifications which could generate conjugate vaccines by fusing OMVs to heterologous antigens (Gerritzen et al., 2017; Cheng et al., 2021). This tumor vaccine has many advantages when compared with conventional vaccines: (1) the antigen retains its natural conformation, (2) the vaccine retains its ability to target specific immune responses, and (3) a single production process can supply multiple vaccines (Micoli and MacLennan, 2020; Aytar Çelik et al., 2022).

### 7.2. OMVs as cancer immunotherapy agents

Cancer therapy is undergoing a paradigm shift toward immunotherapy, with research focused on new ways to activate the host’s anti-tumor immune system. OMVs are major players in this field as they contain multiple immunostimulatory molecules that allow immune cells to recognize and uptake OMVs, thus eliciting systemic humoral and cell immune responses (Zhang et al., 2019; Suri et al., 2023). Additionally, OMVs are highly permeable, are retained, and preferentially accumulate around tumor tissue to induce local immune responses. Moreover, because they are non-replicating particles, they are relatively safer in hosts when compared with live bacteria (Bitto and Kaparakis-Liaskos, 2017). In short, these inherent characteristics of OMVs confer them huge potential as cancer immunotherapeutic agents.

Kim et al. (2017) investigated the potential of *E. coli* OMVs as immunotherapeutic agents for CRC. By directly injecting inactivated *E. coli*-derived OMVs into mice bearing CT26 colon adenocarcinomas, OMVs specifically accumulated at tumor sites and induced long-term antitumor immune responses to eliminate tumors. Mechanistically, OMV-induced antitumor effects were dependent on IFN-γ, as IFN-γ-deficient mice did not induce OMV-mediated immune responses (Kim et al., 2017).

While studies have reported that gut microbiota OMVs induced effective anti-CRC immune responses, therapeutic effect can be further optimized using genetic engineering or combinations with other CRC therapeutics (Chen et al., 2020). Firstly, immune checkpoint inhibitor expression on OMV surfaces, using genetic engineering, may enhance antitumor immune responses because immune checkpoint inhibitors can reverse immunosuppression and eradicate tumors, especially when combined with other treatments (Bagchi et al., 2021; Kaushik et al., 2022). For example, in a mouse CRC model, researchers inserted the ectodomain of programmed death 1(PD1)on the OMVs surface by genetic engineering techniques. The engineered OMV-PD1 can bound to programmed death 1 ligand 1 (PD-L1), accumulated around tumor tissue, and significantly reduced tumor growth *via* immune activation and PD1/PD-L1 axis blockade combined effects (Li et al., 2020b). This study used a new strategy to genetically engineer OMVs for combination CRC therapy. Additionally, OMVs were used as chemotherapeutic drug adjuvants to improve conventional chemotherapy efficacy for CRC. One study showed that *S. typhimurium* OMVs were cytotoxic to the CRC cell line Caco-2 and decreased cell viability when compared with chemotherapies. More importantly, OMVs exhibited more potent cytotoxic effects when combined with chemotherapies, with dose-dependent decreases in cell viability in tumor-bearing mice (Aly et al., 2019). Similarly, in another study, *S. typhimurium* OMVs significantly reduced tumor size and significantly improved tumor growth inhibition as a single immunotherapy or an adjuvant to conventional chemotherapy in mouse models expressing transplanted Ehrlich solid carcinoma (Aly et al., 2021). Thus, *S. typhimurium* OMVs may not only function as promising novel anti-CRC immunotherapies, but may function better as adjuvants to potentiate chemotherapy efficacy in CRC and reduce harmful side effects.

As mentioned above, gut microbiota OMVs may have positive roles in stimulating robust and durable antitumor immune responses and establishing novel therapeutic strategies for CRC.

### 7.3. OMVs as delivery vehicles

Gut microbiota OMVs may also function as drug delivery vehicles in CRC therapy. Their size and natural lipid bilayer structure permits their accommodation of many biomolecules with different properties, and protects these molecules from degradation. Moreover, bioengineering technology allows their targeted delivery without toxicity to peritumor tissue (Liu et al., 2022b).

Studies have shown that OMVs act as carriers of chemotherapeutic drugs, small interfering RNAs (siRNAs), or other agents, increasing their accumulation in tumors. Moreover, targeted transport can be further enhanced by genetically engineering OMVs (Gujrati et al., 2014; Aytar Çelik et al., 2023). Currently, chemotherapy remains an important clinical treatment for CRC. Among commonly used chemotherapeutic agents, 5-fluorouracil (5-FU) is a standard chemotherapy for intestinal cancer, whether single- or combination-chemotherapy. However, these treatment options have serious adverse effects in humans, including low bioavailability and systemic toxicity (Vodenkova et al., 2020; Olguin et al., 2023). In a recent study, *E. coli* OMVs wrapped in conventional nanocarrier mesoporous silica were loaded with 5-FU to treat colon cancer. OMVs delivered 5-FU to the target colon without mononuclear phagocyte system interference. This composite drug carrier exploited ideal OMV biocompatibility, thus achieving better *in vivo* stability, reducing damage to other organs, and achieving better therapeutic effects (Shi et al., 2020). Another study, using OMVs as *in vivo* delivery vehicles, reported targeted siRNA delivery to tumor cells by expressing a targeting ligand on OMVs. Gujrati et al. (2014) used electroporation to load siRNA targeting kinesin spindle protein (KSP) into *E. coli*-derived OMVs and engineered OMVs to express a human epidermal growth factor receptor 2-specific affibody as a tumor-targeting ligand. Systematic siRNA-packaged OMV injection into animal models showed that OMVs inhibited KSP expression, targeted and killed cancer cells in a cell-specific manner, and significantly retarded tumor growth (Gujrati et al., 2014). The authors proposed that bioengineered OMVs had great potential as cell-specific drug-delivery vehicles for treating different cancers.

Therefore, gut microbiota OMVs can be used as delivery vehicles to load chemotherapeutic drugs or other antitumor agents, and provide new strategies for more effective CRC targeted therapies.

### 7.4. OMVs as diagnostic biomarkers

Gut bacterial OMVs may have important roles in CRC diagnostics. OMVs are in essence information delivery carriers secreted by bacteria, and contain nucleic acids, proteins, and/or lipid molecules derived from parental bacteria. Detecting these molecules may characterize intestinal microbiota and metabolite changes in patients. Additionally, when compared with direct gut microbiota detection, OMV biomarkers may avoid interference from partially-inactivated bacteria (Liang et al., 2022).

It is accepted that CRC pathogenesis is closely related to gut microbial dysbiosis. One study detected gut microbiota OMVs from the stool samples of CRC patients and healthy volunteers, and metagenomic profiling showed that the gut microbiome in CRC patients was significantly altered (Kim et al., 2020). Thus, detecting gut microbiota OMVs may highlight relationships between the gut microbiota and CRC pathogenesis.

Bioimaging technology directly reflects tumor growth, metastasis, and drug responses, and is vital for guiding early cancer diagnoses (Gu et al., 2019a). Exogenous bioimaging probes can be designed and anchored onto OMVs for more stable cancer cell imaging (Xue et al., 2022). For instance, based on the fact that the nanoluciferase (Nluc) fuses with the *E. coli* OM lipoprotein SlyB, Chen et al. (2017) successfully loaded Nluc into OMVs and showed that these engineered OMVs emitted distinct luminescent signals both *in vitro* and *in vivo*. Therefore, the further refinement of OMVs as biomarkers may generate more effective CRC diagnostic tools.

## 8. Conclusion and perspectives

As described, gut microbiota OMVs are important mediators of microbial-host communications and regulate host immune responses, gut homeostasis, and metabolism. They have great potential in biomedical applications, such as tumor vaccines, adjuvants, and drug carriers.

While considerable progress has been made in understanding the impact of OMVs on tumors, further studies are required on the role of gut microbiota OMVs in CRC occurrence and development. Currently, most studies on OMVs are at the initial stage of using cell lines without the use of animal models, and the conclusions of these studies may be less convincing or misleading. Therefore, in future research, more clinical trials must be conducted to better elucidate interactions between OMVs and CRC.

Additionally, limitations exist when translating OMVs from the laboratory to the clinic: (1) OMVs purification and isolation techniques are time-consuming and complex laboratory procedures. (2) Some OMVs have toxic effects on hosts as they carry cytotoxic components (e.g., LPS). (3) OMVs have low levels of protective antigen expression. (4) OMVs may carry uncontrollable components which interfere with and suppress host immune responses. To address these issues, we need to further refine the techniques isolating and detecting complex components from OMVs and incorporate the latest technologies (metagenomics, proteomics, metabolomics, and bioinformatics) to guide engineered OMV development and applications toward CRC therapy.

In conclusion, with relevant technology advances and some key challenges addressed, further research will help us understand the complex relationships between OMVs and the host. Undoubtedly, gut microbiota OMVs can become powerful novel cancer treatment tools, and provide new pathways establishing effective CRC prevention, diagnosis, and treatment strategies.

## Author contributions

RM: Writing – original draft, Writing – review & editing. MZ: Writing – review & editing. YJ: Writing – review & editing. XH: Writing – review & editing. MX: Writing – review & editing.
